# Evaluation of immuno-histochemical markers in oral squamous cell carcinoma

**DOI:** 10.6026/973206300191399

**Published:** 2023-12-31

**Authors:** Yadavalli Guruprasad, Priya Singhal, Neha Gupta, Bhavna Jha Kukreja, Bharti Gupta, Pankaj Kukreja, Ramanpal Singh Makkad, Dhaval Niranjan Mehta

**Affiliations:** 1 Department of Dentistry, Vijaynagar Institute of Medical Sciences, VIMS Campus, Cantonment, Ballari - 583104, India; 2 Senior Resident, Department of Dentistry, SMS Medical College, Jaipur, India; 3 Department of Oral Pathology, Microbiology and Forensic Odontology, Dental Institute, Rajendra Institute of Medical Sciences (RIMS), Ranchi, Jharkhand, India; 4 Periodontology Preventive Dental sciences Department College of Dentistry, Gulf Medical University, Ajman UAE; 5 Department of Maxillofacial Surgery and Diagnostic Sciences, College of Dentistry, Jazan University, Jazan 45142, Saudi Arabia; 6 Oral and Maxillofacial Surgery, Department of Biomedical and Dental Sciences, Faculty of Dentistry, Al Baha University, Al Aqiq Campus, Kingdom of Saudi Arabia; 7 Department of Oral medicine and Radiology, New Horizon Dental College and Research, Institute, Sakri, Bilaspur, Chhattisgarh, India; 8 Department of Oral Medicine and Radiology, Narsinbhai Patel Dental college and Hospital, Sankalchand Patel University, Visnagar, Gujarat, India

**Keywords:** Immunohistochemistry, markers, oral squamous cell carcinoma

## Abstract

Evaluation of immuno-histochemical (IHC) markers like p53, p63, PDPN, C-erb-B2, CK19, and VEGF in oral squamous cell carcinoma (OSCCs) is of interest to dentists. Sixty formalin-fixed paraffin embedded tissue blocks from the Department of Oral Pathology, New Horizon Dental College and Research, Institute, Sakri, Bilaspur, Chhattisgarh, India. The conventional IHC method was used to assess the expression of p53, p63, PDPN, C- erb-B2, CK19 and VEGF using the different antibodies. Data shows that P53, p63 had high values of labeling index (LI) of staining while PDPN, C-erb-B2 had low values of LI of staining. The values of LI of staining for CK19, and VEGF were in between the two types of IHCs. Combining the analysis of multiple IHC markers for OSCC can yield precise cancer diagnosis results.

## Background:

The most prevalent type of squamous cell carcinoma of the head and neck is oral cancer, often known as oral squamous cell carcinoma (OSCC) [1,2]. The death rate from this type of tumour has been rising lately. Carcinogens like cigarettes, tobacco and alcohol have been linked to higher amounts of harm to DNA and other health problems as people age progresses [3,4]. The most prevalent type of epithelial-originated oral cancer detected in the cavity of the mouth is epithelial cell carcinoma [5, 6]. Tumour markers are among the most widely utilised techniques in laboratory cancer diagnosis. Combining the analysis of multiple biomarkers can yield more trustworthy and precise cancer diagnosis results [7, 8]. One member of the extensive group of transitional filamentous proteins, which is separated into primary (CK1-CK8) and acidic (CK9-CK23) polypeptides, is cytokeratin 19 [3-7, 9,10,11, 12]. The whole epithelial cell's maintenance as well as the cell cycle's reaction to stress as well as apoptosis depend heavily on CK 19 [2-5, 13,14,15]. The glycoprotein known as vascular endothelial growth factor (VEGF) is homo dimeric. It is a cytokine that attaches to the VEGF ligand on the outer layer of endothelial cells and has the ability to promote angiogenesis. It is an essential multifunctional mechanism in the inflammatory process and healing of wound procedures [10-14].

The most frequent source of genetic changes in human tumours is the tumour suppressor gene p53. This gene produces a protein located in nucleus that is involved in the abolition of gene equilibrium, programmed death of cells or apoptosis, and cell cycle regulation [16,17]. Immunohistochemistry can be used to detect the prolonged half-life of the p53 mutant gene's polypeptide product. Oncogenes have also been identified as a potential cause of squamous cell cancer [18,19]. Among these, C-erb-B's function as a cell membrane glycoprotein linked to epidermal growth elements that function as oncogenes at a higher frequency has been given attention lately. Samples collected from dysplastic tissue have been found to exhibit a rise in C-erb-B2 [20, 21].

Head and neck malignancies have been linked to p53 gene mutations, and dysplastic as well as SCCs specimens have been confirmed to express p53 [22,23]. Additionally, some research indicates that p53-positive cells that originate from hyperplastic lesions convert to dysplastic and cancerous lesions. One sign of malignancy is unchecked cell proliferation. By labelling nuclear antigens linked to division and growth of cells and analyzing it by immuno histochemical methods under a light microscope, this cell multiplication can be identified [24, 25]. In S phase, M phase, G1 phase, and G2 phase of the cell cycle, one non-histone nuclear protein produced is linked to the antigen ki67[12-16]. As so, it will serve as a helpful marker for mitosis and cell division. The occurrence of p53 and ki67 was shown to be significantly correlated, and this has been linked to the proliferative action of cells [13- 18].

It has been demonstrated that oral dysplastic lesions and OSCC both have elevated p63 levels. In addition to controlling cell division and proliferation, p63 and p53 may also be involved in the malignant development from oral leukoplakia [14- 17]. As a result, both proteins ought to be viable biomarker possibilities for oral leukoplakia relapse and carcinoma transformation [18-22]. A transmembrane glycoprotein of the mucin type, PDPN is found extensively in various tissues and cells [12- 16].PDPN is elevated in dermal fibroblast-like cells and basal epidermal keratinocytes in hyperproliferative situations like psoriasis, healing of wounds, or in response to stimuli to inflammation [11- 17]. According to functional research, PDPN encourages the growth of tumours. PDPN has been reported to be excessively expressed in OSCC and to be a valuable biomarker in OL for evaluating the possibility of cancerous transformation [18-22]. Therefore, it is of interest to assess the relationship between p53, p63, PDPN, C-erb-B2, CK19 and VEGF in OSCCs.

## Methods and Materials:

## Specimens of study:

There was total sixty formalin-fixed paraffin embedded tissue blocks from the Department of Oral Pathology, New Horizon Dental College and Research, Institute, Sakri, Bilaspur, Chhattisgarh, India (Table 1). They were divided into four categories. In each of the four categories, the immunohistochemistry expression of p53, p63, PDPN, C-erb-B2, CK19, and VEGF was evaluated. Formalin-fixed paraffin embedded tissue blocks collected from records of oral pathology department were subjected to a retrospective cross-sectional immunohistochemistry examination. 45 blocks of histo-pathologically proven OSCC with histological diagnosis were included in the study that included fifteen tissue blocks of poorly-differentiated oral carcinoma, fifteen tissue blocks of well-differentiated oral carcinoma, and fifteen tissue blocks of moderately-differentiated oral carcinoma. 15 specimens of of normal oral mucosa (NM) were examined.

The conventional IHC method was used to assess the expression of p53, p63, PDPN, C- erb-B2, CK19, and VEGF using the different antibodies. The antibody for different IHC markers were as follows: p53= anti p53 antibody, p63= anti p63 antibody, PDPN= antibody PDPN, C- erb-B2= antibody C- erb-B2, CK19= antibody CK 19, VEGF= antibody VEGF. Positive staining was demonstrated by the existence of a brown-colored final product at the target antigen location. Nuclear staining varied in intensity across all cases. The staining intensity was examined to determine the degree of stain absorption. On each slide, ten randomly chosen fields were magnified at a magnification of x40. The following scale was used to rate the staining intensity of each section [11- 12]. No stain = 0, mild stain= 1, moderate stain = 2, intense stain= 3. After scanning the complete section of the epithelium, the area of coloured epithelial cells was measured in order to ascertain the expression profile and the amounts of protein accumulation in the epithelial layers.:[17] 0% = 0, 25% = 1,25%-49% = 2 ,50%-74% = 3, 75%-100% =4. After the slides were viewed at x40 magnification using an Olympus CX21 light microscope, illustrative photomicrographs were made of each slide in five different hotspot areas in order to calculate the labelling index (LI). After that, an image processing programme called ImageJ (http://imagej.nih.gov/ij/) was used to analyse the photomicrographs. The total number of tumour cells in each slide was computed until a minimum of 400 cells were attained, i.e addition of the denominators (x). The percentage of IHC positive tumour cells per hot spot (A) was also determined [8]. The following formula was used to compute LI [9].

LI % =Ax100/Nooftumourcells

## Statistical analysis:

By dividing the number of positive cells by the total number of cells counted in the slide, the LI, or percentage of positive cells, for each slide in each category was determined. To determine whether there is a significant difference in the mean LI between the categories for each antibody, the Kruskal Wallis one-way ANOVA test was performed. Pairwise comparisons of LI of p53, p63, PDPN, C-erb-B2, CK19, and VEGF between the categories were performed applying the Mann-Whitney U test in alongside the one way ANOVA. The Spearman rank correlation test was used to compare the LIs of p53, p63, PDPN, C-erb-B2, CK19, and VEGF for each group in order to determine whether there was a relationship. To determine whether there is a relationship between the groups' patterns or distributions, LI, and staining intensities for each antibody, the χ² test was employed. When the P-value was less than 0.05, it was deemed significant.

## Results:

The intensity of staining for different IHC markers p53, p63, PDPN, C-erb-B2, CK19, and VEGF was greater in all three groups of OSCC (poorly differentiated OSCC, moderately differentiate OSCC and well differentiated OSCC) when compared with normal mucosa. The intensity was high in well differentiated OSCC as compared to other categories of OSCC. The intensity of staining was in order of well differentiated OSCC> moderately differentiated OSCC> poorly differentiated OSCC>Normal mucosa. The findings were statistically significant (p=0.001). When there was comparison between different IHCs regarding intensity of staining then P53, p63 had maximum intensity of staining while PDPN, C-erb-B2 had minimum intensity of staining. The intensity of staining for CK19, and VEGF was in between the two types of IHCs (Table 2). The area of staining for different IHC markers p53, p63, PDPN, C-erb-B2, CK19, and VEGF was greater in all three groups of OSCC (poorly differentiated OSCC, moderately differentiate OSCC and well differentiated OSCC) when compared with normal mucosa. The area was high in poorly differentiated OSCC as compared to other categories of OSCC. The area of staining was in order of poorly differentiated OSCC> moderately differentiated OSCC> well differentiated OSCC. The findings were statistically significant (p=0.001). When there was comparison between different IHCs regarding area of staining then P53, p63 had maximum area of staining while PDPN, C-erb-B2 had minimum area of staining. The area of staining for CK19, and VEGF was in between the two types of IHCs (Table 3).

The values of LI for different IHC markers p53, p63, PDPN, C-erb-B2, CK19, and VEGF was greater in all three groups of OSCC (poorly differentiated OSCC, moderately differentiate OSCC and well differentiated OSCC) when compared with normal mucosa. The values of LI were high in poorly differentiated OSCC as compared to other categories of OSCC. The values of LI of staining was in order of poorly differentiated OSCC> moderately differentiated OSCC> well differentiated OSCC>Normal mucosa. The findings were statistically significant (p=0.001). When there was comparison between different IHCs regarding values of LI of staining then P53, p63 had maximum values of LI of staining while PDPN, C-erb-B2 had minimum values of LI of staining. The values of LI of staining for CK19, and VEGF were in between the two types of IHCs (Table 4).

## Discussion:

There is increase in LI values for different IHC markers in poorly differentiated OSCC as compared to moderately differentiated and well differentiated OSCC [12-16]. Moreover, the values of LI were greater in p53, p63 IHC markers. Some studies has findings different from our study where they found increased LI values for IHC markers other than p53 and p63[17-23].The most common genetic alteration found in human cancers is caused by the p53 tumour suppressor gene. This gene produces in a protein that is found in the nucleus and is involved in cell cycle regulation, programmed cell death, and the elimination of gene equilibrium [11- 14]. The polypeptide product of the p53 mutant gene has an extended half-life, which can be identified by immunohistochemistry. Another factor that has been linked to squamous cell carcinoma is oncogenes [15, 17]. Among these, attention has recently focused on the role of C-erb-B as a glycoprotein found in cell membranes that are connected to epidermal growth elements that act as oncogenes more frequently. It has been discovered that samples taken from dysplastic tissue contain an increase in C-erb-B2 [18-24]. Our study's results were consistent with those of other studies. These investigations discovered that, in comparison to moderately and highly differentiated OSCC; there is an increase in the stained area for various IHC markers in poorly differentiated OSCC [13-19] Furthermore, the IHC markers for p53 and p63 showed a larger stained region. Some studies observed higher staining for IHC markers other than p53 and p63, which is distinct from the results of our study [14-20].

Mutations in the p53 gene have been associated with head and neck cancers, and p53 expression has been verified in specimens from dysplastic and SCCs [16-18]. Furthermore, some studies show that cells that are positive for p53 from hyperplastic lesions might develop into dysplastic and malignant lesions. Unchecked cell proliferation is one indicator of cancer [18-22]. This cell multiplication can be recognised by labelling nuclear antigens associated with cell division and growth and examining it immuno-histo-chemically under a light microscope [23,25, 26]. One non-histone nuclear protein generated in the cell cycle is associated with the antigen ki67 in the S, M, G1, and G2 phases. As such, it will be a useful indicator of cell division and mitosis [24, 27,28].

Significant correlations between the expression of p53 and ki67 have been observed, and they have been connected to the proliferative activity of cells. It has been shown that p63 levels are raised in both OSCC and oral dysplastic lesions [10- 16]. P63 and p53 may have a role in the malignant progression of oral leukoplakia in addition to regulating cell division and proliferation. Therefore, both proteins should be promising candidates for oral leukoplakia relapse and carcinoma transformation biomarkers [17-21]. PDPN is a transmembrane glycoprotein of the mucin type that is widely distributed in a wide range of tissues and cells. Dermal fibroblast-like cells and basal epidermal keratinocytes have high PDPN in hyper-proliferative conditions such as psoriasis, wound healing, or in response to inflammatory stimuli [22-26].

Functional research indicates that PDPN promotes the growth of tumours. It has been noted that PDPN is overexpressed in OSCC and that it is a useful biomarker in OL for assessing the likelihood of malignant transformation [21- 25]. Cytokeratin 19 is one of the several transitional filamentous proteins that are divided into primary (CK1-CK8) and acidic (CK9-CK23) polypeptides. CK 19 is essential for the upkeep of the whole epithelial cell as well as the cell cycle's response to stress and apoptosis. Vascular endothelial growth factor (VEGF) is a homo dimeric glycoprotein [12- 17]. This cytokine can stimulate angiogenesis by adhering to the VEGF ligand on the surface of endothelial cells. It is a crucial multifunctional mechanism in the wound-healing and inflammatory processes [23- 27].

Recently, the death rate from OSCC has increased. As people age, carcinogens such as alcohol, tobacco, and cigarettes have been related to increased levels of DNA damage and other health issues [13,14]. Epithelial cell carcinoma is the most common kind of epithelial-originated oral cancer found in the oral cavity [15, 16]. One of the most popular methods used in laboratory cancer diagnosis is the use of tumour markers. Results from the combination of numerous biomarker analyses for cancer diagnosis can be more accurate and reliable.

## Conclusion:

Data shows that IHC markers like p53, p63, PDPN, C-erb-B2, CK19, and VEGF are useful for the diagnosis of oral squamous cell carcinoma.

## Figures and Tables

**Table 1:** Distribution of study specimens

**Category**	**No of specimens**
Poorly differentiated OSCC	15
Moderately differentiated OSCC	15
Well differentiated OSCC	15
Normal mucosa	15

**Table 2:** Comparison of for different IHC markers regarding intensity of staining between the study groups

	**Well differentiated OSCC**	**Moderately Differentiated OSCC**	**Poorly differentiated OSCC**	**Normal Mucosa**	**P value**
p53	2.821±0.117	2.972 ±0.213	3.001±0.113	1.722±0.919	0.001*
p63	2.800±0.627	2.900±0.117	2.989±0.627	1.100±0.738	0.001*
PDPN	2.349±0.887	2.450±0.998	2.849±0.321	1.533±0.696	0.001*
C-erb-B2	2.411±0.616	2.600±0.505	2.689±0.312	1.143±0.738	0.001*
CK19	2.671±0.112	2.762±0.234	2.863±0.410	1.788±0.818	0.001*
VEGF	2.521±0.627	2.700±0.738	2.911±0.434	1.211±0.849	0.001*
P value	0.341				
*indicates statistically significant difference p<0.05.

**Table 3:** Comparison of different IHC markers regarding area of staining between the study groups for

	**Poorly differentiated OSCC**	**Moderately Differentiated OSCC**	**Well differentiated OSCC**	**Normal Mucosa**	**P value**
p53	2.721±0.118	2.872 ±0.102	3.101±0.002	1.622±0.808	0.001*
p63	2.711 ± 0.416	2.811±0.006	2.889±0.516	2.211±0.748	0.001*
PDPN	2.238±0.895	2.341±0.887	2.738 ±0.210	1.422±0.587	0.001*
C-erb-B2	2.300±0.505	2.511±0.505	2.578±0.201	2.043±0.738	0.001*
CK19	2.560±0.001	2.651±0.123	2.752±0.301	1.677±0.707	0.001*
VEGF	2.410 ±0.516	2.811±0.627	2.800±0.323	1.311±0.738	0.001*
P value	0.672				
*indicates statistically significant difference p<0.05.

**Table 4:** Values of Labelling Index for different IHCs in different study groups

	**Well differentiated OSCC**	**Moderately Differentiated OSCC**	**Poorly differentiated OSCC**	**Normal Mucosa**	**P value**
p53	42.260 ± 1.327	44.371 ± 4.325	45.260 ± 5.436	9.53± 4.109	0.001*
p63	43.532± 8.372	44.002± 2.212	44.532± 9.483	19.442± 2.882	0.001*
PDPN	18.115 ±2.105	19.015 ±2.003	20.026 ±3.214	7.31± 2.017	0.001*
C-erb-B2	20.121 ± 07.356	21.221 ± 08.467	22.300 ± 3.344	17.220± 2.660	0.001*
CK19	29.026± 2.103	30.026± 1.013	31.037± 3.214	7.31± 2.017	0.001*
VEGF	31.101 ± 8.352	32.310 ± 3.356	33.421 ± 9.467	17.220± 2.660	0.001*
P value	0.472				
*indicates statistically significant difference p<0.05.
